# O.1.2-10 Intergenerational co-production to increase physical activity and social connectivity for health in the GOALD project (Generating Older Active Lives Digitally)

**DOI:** 10.1093/eurpub/ckad133.097

**Published:** 2023-09-11

**Authors:** Anna C Whittaker, Simone A Tomaz, Hannah Bradwell, John Ritchie, Katie Edwards, Leonie Cooper, Catherine Hennessy, Ray Jones, Richard Haynes, Gemma C Ryde, Pete Coffee, on behalf of the GOALD Research Team, the GOALD Advisory Group

**Affiliations:** University of Stirling, UK; University of Stirling, UK; University of Plymouth, UK; University of Stirling, UK; University of Plymouth, UK; University of Plymouth, UK; University of Stirling, UK; University of Plymouth, UK; University of Stirling, UK; University of Glasgow, UK; Heriot Watt University, UK; A.C.Whittaker@stir.ac.uk; University of Stirling, UK; University of Plymouth, UK

## Abstract

**Purpose:**

The Generating Older Active Lives Digitally (GOALD) project aimed to explore how to use digital technology to keep older people physically active and socially connected for health and wellbeing. This project took a novel inclusive interdisciplinary approach working with a range of collaborating organisations to review and co-design digital technologies that support social connectedness, through physical activity, and sport reminiscence. This presentation presents the outcomes from Phase 1 where researchers engaged with an advisory group to determine key challenges and potential outcomes of intergenerational engagement with digital technology for health and wellbeing.

**Methods:**

Participants were 28 individuals with experience in working with older people (range: 1-41 years) across different contexts (e.g., nursing care, social work, family care) and with some themselves being older (age range: 32-79 years). Over eight months, advisory group members were invited to ten 1hr meetings facilitated by GOALD researchers to help develop data collection measures, and address recruitment issues for future co-production and design plans. Recordings were transcribed and subjected to thematic content analysis.

**Results:**

Challenges identified at advisory group meetings worked as a proxy for anticipated challenges as the GOALD project unfolded. Examples of key challenges were continuing COVID-19 restrictions in England and Scotland, internet disruptions, care home staffing, and family responsibilities. The shared expertise from the advisory group through their working and lived experiences helped to direct and refine the next steps of the GOALD project, including appropriate survey design, awareness of the contextual realities of research in care homes, and appropriate consideration of people with different abilities.

**Conclusions:**

Lessons learned about how challenges such as the care home context, timing, technical support, and competing priorities influence co-produced research helps to inform researchers on how to approach intergenerational and/or co-productive research methods and design of tools to enhance older adult health and wellbeing through physical activity and sport.

**Support/Funding Source:**

This project has received funding from the UKRI and ESRC Healthy Ageing Social Behavioural and Design Research Programme (SBDRP) grant No ES/V016113/1. https://www.plymouth.ac.uk/research/centre-for-health-technology/goald

